# Exploring the application and mechanism of sodium hyaluronate in cryopreservation of red blood cells

**DOI:** 10.1016/j.mtbio.2021.100156

**Published:** 2021-11-10

**Authors:** Xiangjian Liu, Yuying Hu, Yuxin Pan, Meirong Fang, Zhen Tong, Yilan Sun, Songwen Tan

**Affiliations:** Xiangya School of Pharmaceutical Sciences, Central South University, Changsha, Hunan, 410013, China

**Keywords:** Transfusion, Cryopreservation, Sodium hyaluronate, Red blood cells, Cryoprotectant, Mathematical model

## Abstract

The cryopreservation of red blood cells (RBCs) is essential for transfusion therapy and maintaining the inventory of RBCs units. The existing cryoprotectants (CPAs) have many defects, and the search for novel CPAs is becoming a research hotspot. Sodium hyaluronate (SH) is polymerized from sodium glucuronate and *N*-acetylglucosamine, which has good water binding capacity and biocompatibility. Herein, we reported for the first time that under the action of medium molecular weight sodium hyaluronate (MSH), the thawed RBCs recovery increased from 33.1 ​± ​5.8% to 63.2 ​± ​3.5%. In addition, RBCs functions and properties were maintained normally, and the residual MSH could be removed by direct washing. When MSH was used with a very low concentration (5% v/v) of glycerol (Gly), the thawed RBCs recovery could be increased to 92.3 ​± ​4.6%. In general, 40% v/v Gly was required to achieve similar efficiency. A mathematical model was used to compare the performance of MSH, PVA and trehalose in cryopreservation, and MSH showed the best efficiency. It was found that MSH could periodically regulate the content of intracellular water through the “reservoir effect” to reduce the damages during freezing and thawing. Moreover, MSH could inhibit ice recrystallization when combined with RBCs. The high viscosity and strong water binding capacity of MSH was also conducive to reducing the content of ice. This works points out a new direction for cryopreservation of RBCs and may promote transfusion therapy in clinic.

## Highlights

• MSH improved the RBCs recovery in cryopreservation.
• MSH can be removed directly after thawing.
• The properties and functions of RBCs were protected by MSH.
• High RBCs recovery is found using MSH with 5% v/v glycerol.
• The mathematical model is studied for the cryopreservation.
• The mechanism is proposed for cryopreservation using MSH.

## Graphical abstract

**Figure:**
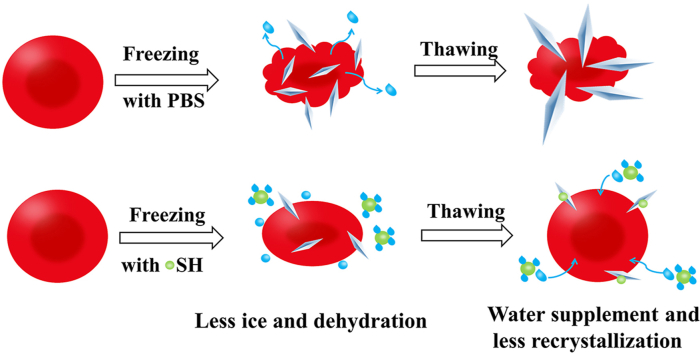

## 1 Introduction

Transfusion of red blood cells (RBCs) plays an important role in modern medicine. Diseases such as traumas, leukemia, hemolytic anemia and so on can be treated with transfusion [1]. It was reported that over 100 million units of donated RBCs were collected worldwide in 2018 [2], while more than 15 million units were consumed annually in the United States alone [3]. Such a huge demand for RBCs requires good storage methods. The currently collected RBCs are generally stored at 4 ​°C for up to 42 days [4]. However, RBCs will undergo hemolysis, morphological changes and accumulation of metabolites when stored for over 21 days [5,6], and the use of long-term stored RBCs may increase the risk of death [7]. The cryopreservation technology provides a new method for RBCs storage. Below 0 ​°C, the metabolism of RBCs will be significantly reduced or even stopped [8], which can alleviate many adverse reactions during storage.

Cells will suffer osmotic damage and mechanical damage during cryopreservation [9]. Osmotic damage refers to the increase of osmotic pressure caused by the freezing of solvents, which leads to cell osmotic dehydration. Mechanical damage is the rupture of cell membranes due to the penetration of ice crystals. In particular, large ice formed by ice recrystallization during the thawing process may be fatal to cells. To reduce the damages during cryopreservation, appropriate cryoprotectants (CPA) must be added [10]. Glycerol (Gly) is the most common CPA for cryopreservation of RBCs [11]. Gly can replace part of the water in RBCs, which can reduce the formation of intracellular ice crystals and alleviate the dehydration of the cells during cryopreservation [12,13]. To achieve the ideal efficiency, a high concentration of Gly (20–40%) must be used, and the temperature must be decreased to −196 ​°C at a very fast rate (∼100 ​°C/min) [14]. This cryopreservation method using high concentration CPA and rapid cooling is known as vitrification [15]. However, Gly can cause side effects such as deformation of RBCs and hemolysis, so Gly must be removed sufficiently before RBCs transfusion to the patient [16]. The removal process requires multiple steps of washing with a gradient concentration of Gly [17], which is cumbersome and expensive. Nevertheless, the washing process still causes ∼15% RBCs hemolysis and ∼1% Gly residue in the cells [11]. Therefore, the search for novel, non-side-effect, and easy-to-remove CPA is becoming a research hotspot. Block copolymer worms [13], metal-organic framework nanoparticles [12], betaine [3], trehalose [14], proline [9], etc. have been developed to replace Gly or reduce the amount of Gly in the cryopreservation of RBCs.

**Fig. 1:**
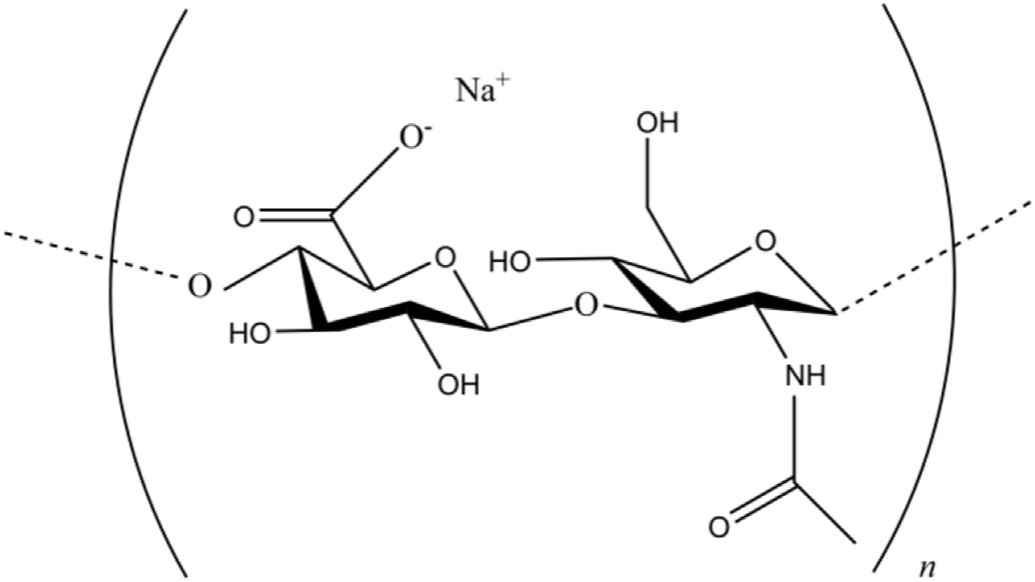
The molecular structure of SH.

Sodium hyaluronate (SH) is polymerized from sodium glucuronate and *N*-acetylglucosamine, with molecular weights up to millions (Fig. 1). SH exists in the extracellular matrix of synovial fluid and connective tissue [18] with no immunogenicity [19] and has extensive application in medicine. For example, SH can be used as artificial tear [20], joint lubricant [21], skin conditioning agents [22], and can also accelerate the healing process in tooth sockets of rats [23], help treat bladder diseases [24]. The properties of hyaluronic acid (HA) are similar to SH, and HA was also widely used in biology and medicine such as tissue engineering and cancer treatments [25]. In cryopreservation, SH could replace newborn calf serum in embryo freezing media [26], and reduce the concentrations of Gly in the cryopreservation of embryo [27]. HA has also been reported as an additive for cryopreservation of germ cells. When HA was added to the extender, the mobility of ram [28] and human [29] sperm after cryopreservation was improved. However, SH (HA) has not been used for cryopreservation alone, and the mechanism of SH (HA) in cryopreservation has not been discovered.

Herein, we for the first time explored the feasibility of SH in cryopreservation of RBCs and put forward the mechanism of SH in cryopreservation in this study. The results have shown that satisfactory thawed RBCs recovery could be achieved with medium molecular weight SH (MSH). When MSH was used with a very low concentration of Gly (5% v/v), the thawed RBCs recovery could reach more than 90%. Due to the strong viscosity and water binding capacity of MSH, the formation of intracellular ice was reduced and the vitrification was promoted. Ice recrystallization was also inhibited when MSH was activated by RBCs. We believe the study can provide a new perspective for cryopreservation and contribute to the clinical application of transfusion.

## 2 Materials and methods

### 2.1 Materials

Sheep RBCs were obtained from Guangzhou Hongquan Co., Ltd. High, medium, low and ultra-low molecular weight sodium hyaluronate (HSH, MSH, LSH and ULSH) were obtained from Shanghai Yuanye Bio-Technology Co., Ltd. Gly, sucrose and Victoria blue B (VBB) were all obtained from Sinopharm Chemical Reagent Co., Ltd. 0.01 ​M Phosphate-buffered saline (PBS) was obtained from Guangzhou Howei Pharma Tech Co.,Ltd. Milli-Q water (18.2 ​MΩ ​cm^−1^) was used in all experiments.

### 2.2 RBCs preparation

RBCs were collected in centrifuge tubes with PBS and shaken to mix evenly. Then RBCs were centrifuged at 4000 ​rpm for 3 ​min, and the supernatant that contained anticoagulant, white blood cells and plasma was removed. the above operation twice was repeated three times to obtain washed RBCs.

### 2.3 Biocompatibility test

Four different molecular weights of SH (HSH, MSH, LSH, and ULSH) were formulated in PBS at 1 ​mg/mL. Equal amounts of washed RBCs (100 ​μL) were added into PBS and SH solutions as the control group and experimental groups, respectively. The samples were stored at 4 ​°C and centrifuged at 4000 ​rpm for 3min after 24 ​h. The absorbance of the supernatant was measured at 450 ​nm (SHIMADZU, UV2600, Japan). The RBCs recovery can be calculated by the following equations [9].

(1) $$\text{Hemolysis\%}=\frac{A-A_{0}}{A_{1}-A_{0}}\times 100\%$$

(2) RBCs ​recovery ​%=1−Hemolysis(%)

where A is the absorbance of the measured sample, A_0_ is the absorbance of the RBCs in PBS, and A_1_ is the absorbance of RBCs in deionized water.

### 2.4 Scanning electron microscopy (SEM) analysis

After incubation in the four different molecular weights of SH for 24 ​h by the above method, the RBCs were fixed in 2.5% glutaraldehyde for 12 ​h. Then the glutaraldehyde was removed and RBCs were washed with PBS three times to remove the residual glutaraldehyde. The RBCs were fixed again in 1% osmic acid for 1 ​h. Subsequently, the osmic acid was removed and RBCs were washed with PBS three times. The RBCs were dehydrated in 30%, 50%, 70%, 80%, and 95% alcohol once for 15min, and 100% alcohol twice for 20 ​min. Then the RBCs were incubated in a 1:1 solution of ethanol and isoamyl acetate for 30min and were incubated in pure isoamyl acetate for 1 ​h. After supercritical drying, the RBCs were gold-coated and photographed under scanning electron microscopy (Hitachi, SU8010, Japan).

### 2.5 Cryopreservation of RBCs

All the Gly or different molecular weights SH solutions were prepared in PBS at the desired concentration. The combined solution of Gly and MSH was also prepared as described above. Equal washed RBCs (100 ​μL) were mixed with 5 ​mL of the prepared solution and PBS as the experimental group and the control group, respectively. After incubation at room temperature for 30min, the samples were immersed directly into liquid nitrogen until completely frozen as the fast freezing. For the slow freezing, the samples were stored at 4 ​°C for 2 ​h and then transfer to −20 ​°C for 8 ​h. The frozen RBCs were rewarmed in a 37 ​°C water bath. After thawing the samples were centrifuged at 4000 ​rpm for 3 ​min. The absorbance of the supernatant was measured at 450 ​nm. The thawed RBCs recovery can be calculated by equations (1), (2).

### 2.6 Gel permeation chromatography (GPC) test

GPC (Agilent, 1260, USA) was used to analyze the molecular weight distribution. The MSH was prepared as a 2 ​mg/mL and filtered through a 0.22 ​μm microporous membrane. A 50 ​μL sample was taken for detection. 0.2 ​M NaNO_3_ and 0.01 ​M NaH_2_PO_4_ were prepared in pure water to be used as mobile phase, the flow rate was 1 ​mL/min and the temperature was 35 ​°C. The detector was PL-GPC 50 (RI) and polyethylene glycol (PEG) as the standard. The molecular weight distribution of the sample was determined by the elution time.

### 2.7 RBCs morphology observation

A 5 ​μL of washed RBCs was dropped on the end of the glass slide and smoothly push to the other end. A thin layer of RBCs would be left on the glass slide. Then the glass slide was placed under the microscope, and the morphology of RBCs was observed in a clear, non-overlapping area. Pictures were taken by the camera (Huitong, E31S PM, China) on the microscope.

### 2.8 Splat assay

The samples of 1 ​mg/mL MSH and PBS were prepared according to the previous methods. In particular, for the groups of MSH ​+ ​RBCs and PBS ​+ ​RBCs, 400 ​μL washed RBCs were added to 5 ​mL of the above samples to give a hematocrit content of 8%. Then a microscope slide was placed on dry ice to pre-cool to −78 ​°C. A 6 ​μL sample was dropped from a height of about 1.5 ​m onto the glass slide to be instantly dispersed and frozen to a thin layer. Then the microscope slide was quickly moved into a cold stage (Huitong, LTM-190H, China) that maintained at −8 ​°C. The sample was annealed for 20min to allow sufficient time for ice recrystallization. The ice crystals were observed with a polarizing microscope (Huitong, XPF-550, China), and the mean largest grain size (MLGS) was obtained by calculating the mean size of the ten largest crystals in the field of view. MLGS is an index for quantitative analysis of ice recrystallization inhibition (IRI) activity [30].

### 2.9 Sucrose sandwich assay

Enough sucrose was added to the sample to achieve a concentration of 60% wt. Then a 6 ​μL sample was put into two slides and the excess liquid was removed. The slides were immersed in liquid nitrogen for quick freezing, and then moved into a cold stage and annealed at −8 ​°C for 50min. The ice growth was observed under the microscope and images were taken every 2min.

### 2.10 Zeta potential test

A 5 ​μL aliquot of RBCs was added to 1 ​mL 5% glucose solution, and the zeta potential of MSH and RBCs were tested (Malvern, Zetasizer Nano, UK). The number of runs was set as 20, and the number of repeats was set as 3.

### 2.11 Fluorescence analysis

Fluorescence analysis was operated according to the method mentioned before [31]. Fluorescein isothiocyanate (FITC)-labeled SH was dissolved in 0.9% NaCl solution to reach a concentration of 5 ​mg/mL. For the SH ​+ ​RBCs group, enough RBCs were added to give a hematocrit content of 37.5%. Seven droplets of each group were formed onto a glass slide. The droplets were dried under 40% humidity for 5 ​min. Then the glass slide was washed with Milli-Q water and dried with N_2_ gas. A fluorescence microscope (Nikon, ECLIPSE Ti, Japan) was used to observe the trace of remaining droplets, and ImageJ was used to count the fluorescence intensity.

### 2.12 Differential scanning calorimetry (DSC) test

DSC was used to investigate the water binding capacity of MSH. Accurate weight samples (∼15 ​mg) were placed in crucibles. Then they were sealed and transferred to the calorimeter sample chamber (Beijing Henven, HSC-4, China). The samples were cooled from room temperature to −50 ​°C at −3 ​°C/min, held for 5 ​min, and then rewarmed to 15 ​°C at 1 ​°C/min. The heat flow (w/g) was monitored. During freezing, the solutions would be supercooled, which could cause considerable deviation in the measurement. Therefore, the heat flow of the melting process was selected for analysis. The onset temperature of the melting heat flow was taken as the freezing point, and the total water content (w_tc_), the frozen water content (w_f_) and the bound water content (w_b_) could be calculated by the following equations [3]:

(3) $${\text{w}}_{\text{tc}}={\text{m}}_{\text{w}}/\text{m}$$

(4) $${\text{w}}_{\text{f}}=\Delta \text{H}/\Delta {\text{H}}_{\text{w}}$$

(5) $${\text{w}}_{\text{b}}={\text{w}}_{\text{tc}}-{\text{w}}_{\text{f}}$$

where m_w_ and m represent the water mass and the total mass of each sample, respectively, ΔH and ΔH_w_ are the melting enthalpies of each sample and pure water respectively, determined by integration from the onset temperature to the end temperature of the heat flow.

### 2.13 Viscosity test

The Ubbelohde viscometer was washed, dried and put vertically. Then the samples were placed in the viscometer and the time that samples flow out was recorded. The viscosity of pure water at normal temperature and pressure was taken as 1.002 ​× ​10^−3^ ​Pa ​s [32], and the viscosity of the samples could be calculated by the following equation [33]:

(6) $$\frac{\eta }{\eta_{0}}=\frac{\text{t}}{t_{0}}$$

where η and η_0_ are the viscosity of the measured samples and pure water, respectively, t and t_0_ are the time that measured samples and pure water flows through Ubbelohde viscometer, respectively.

### 2.14 CPAs removal by directly washing RBCs

The thawed RBCs were centrifuged at 4000 ​rpm for 3min to initially remove CPAs by removing the supernatant. Then 5 ​mL of PBS was added to the samples and mixed evenly to diffuse the residual CPAs into the solvent. To adequately remove CPAs, the samples were centrifuged at 4000 ​rpm for 3 ​min again and the RBCs recovery after washing was calculated according to the previous method. Then the supernatant was removed and the above operation was repeated once to prevent CPAs residue.

### 2.15 Erythrocyte sedimentation rate (ESR) analysis

Westergren method was used to determine the ESR of RBCs [34]. A 50 ​μL aliquot of washed RBCs after cryopreservation was added in 2 ​mL PBS. The sample was transferred to a Westergren tube and the level of the sample was controlled to the 0-scale mark. A 50 ​μL aliquot of fresh RBCs was treated in the same way as the control group. The ESR of the samples was obtained at 1, 4, 7 and 10 ​h.

### 2.16 Osmotic fragility

The osmotic fragility of RBCs was measured using a stepwise dilution of 1% NaCl ranging from 0.1% to 1%. Then 7.5 ​μL washed RBCs after cryopreservation were incubated with 500 ​μL various dilution buffers for 30min at room temperature. Each sample was centrifuged at 4000 ​rpm for 3min. The absorbance of the supernatant was measured at 450 ​nm, and the hemolysis was calculated by equation (1).

### 2.17 Na^+^/K^+^-ATPase, Ca^2+^/Mg^2+^-ATPase and catalase activities

The enzymatic activities assays were performed with fresh RBCs and washed RBCs after cryopreservation. The ATPases and catalase activities were measured using the inorganic phosphorus method (Nanjing Jiancheng, A070-5-4, China) and the ultraviolet method (Nanjing Jiancheng, A007-2-1, China), respectively. The operation was carried out following the kit instructions.

### 2.18 Mathematical model analysis

Technique for Order Preference by Similarity to an Ideal Solution (TOPSIS) model was used to evaluate the advantages of MSH compared with trehalose and PVA [35,36]. A decision matrix **A** consisting of m alternatives and n criteria was created and denoted by matrix (7). For the convenience of description, we let *M* ​= ​{1,2, …,m} and *N* ​= ​{1,2, …,n}; *i* ​∈ ​*M* and *j* ​∈ ​*N*. Then each attribute value ${\text{a}}_{ij}$ in initial decision matrix **A** was normalized into a corresponding element ${\text{x}}_{ij}$ in the normalized decision matrix **X** by equations (8), (9), (10). The positive ideal solutions (PIS) X^+^ and negative ideal solutions (NIS) X^−^ were determined by the following equations (11), (12), respectively. The distance of each alternative from PIS (D^+^) and that from NIS (D^−^) was given as equations (13), (14). The closeness coefficient of the *i*th alternative S_*i*_ with respect to the ideal solutions was determined as equation (15), and it's proportional to the efficiency of CPAs.

(7) $$\text{A}={\left(a_{ij}\right)}_{m\times n}=\left(\begin{array}{llll} a_{11} & a_{12} & \cdots & a_{1n}\\ a_{21} & a_{22} & \ldots & a_{2n}\\ \vdots & \vdots & \vdots & \vdots \\ a_{m1} & a_{m2} & \ldots & a_{mn} \end{array}\right)$$

(8) $$\text{X}={\left(x_{ij}\right)}_{m\times n}=\left(\begin{array}{llll} x_{11} & x_{12} & \cdots & x_{1n}\\ x_{21} & x_{22} & \ldots & x_{2n}\\ \vdots & \vdots & \vdots & \vdots \\ x_{m1} & x_{m2} & \ldots & x_{mn} \end{array}\right)$$

where

(9) $$x_{ij}=\frac{a_{ij}}{\sqrt{\sum_{i=1}^{m}{\left(a_{ij}\right)}^{2}}},\;for\;benifit\;attribute\;a_{ij},\;i\in N$$

and

(10) $$x_{ij}=\frac{M_{j}-a_{ij}}{\sqrt{\sum_{i=1}^{m}{\left(M_{j}-a_{ij}\right)}^{2}}},\;\text{for}\;\text{cost}\;\text{attribute}\;a_{ij},\;i\in N,\;\text{where}\;{\text{M}}_{j}=\max_{1\leq \text{i}\leq \text{m}}\left\{a_{ij}\right\}\left(j\in N\right)$$

$${\text{X}}^{+}=\left({\text{X}}_{1}^{+},\;{\text{X}}_{2}^{+},\ldots {\text{X}}_{\text{n}}^{+}\right)$$

(11) $$=\left(\max\left\{x_{11},\;x_{21},\;\ldots ,\;x_{\text{m}1}\right\},\;\max\left\{x_{12},\;x_{22},\;\ldots ,\;x_{\text{m}2}\right\}\;,\;\ldots \;,\;\max\left\{x_{1\text{n}},\;x_{2\text{n}},\;\ldots ,\;x_{\text{mn}}\right\}\right)$$

$${\text{X}}^{-}=\left({\text{X}}_{1}^{-},\;{\text{X}}_{2}^{-},\ldots ,{\text{X}}_{\text{n}}^{-}\right)$$

(12) $$=\left(\min\left\{x_{11},\;x_{21},\;\ldots ,\;x_{\text{m}1}\right\},\;\min\left\{x_{12},\;x_{22},\;\ldots ,\;x_{\text{m}2}\right\},\;\ldots \;,\;\min\left\{x_{1\text{n}},\;x_{2\text{n}},\;\ldots ,\;x_{\text{mn}}\right\}\right)$$

(13) $${\text{D}}_{i}^{+}=\sqrt{\sum_{j=1}^{n}{\left(X_{j}^{+}-x_{ij}\right)}^{2}},\;i\in M$$

(14) $${\text{D}}_{i}^{-}=\sqrt{\sum_{j=1}^{n}{\left(X_{j}^{-}-x_{ij}\right)}^{2}},\;i\in M$$

(15) $${\text{S}}_{i}=\frac{{\text{D}}_{i}^{-}}{{\text{D}}_{i}^{-}+{\text{D}}_{i}^{+}},\;i\in M$$

### 2.19 Data analysis

All statistics and calculations are determined by Origin Pro 2017. The results of RBCs experiments are represented by the mean ​± ​standard deviation of three independent experiments. Error bar represents the standard deviation in Figures. Statistical significance determination used the independent-samples *t*-test. A *p*-value less than 0.05 was considered statistically significant. ∗ indicates *p* ​< ​0.05, ∗∗ indicates *p* ​< ​0.01, ∗∗∗ indicates *p* ​< ​0.001.

## 3 Results and discussion

### 3.1 The biocompatibility of SH

**Fig. 2:**
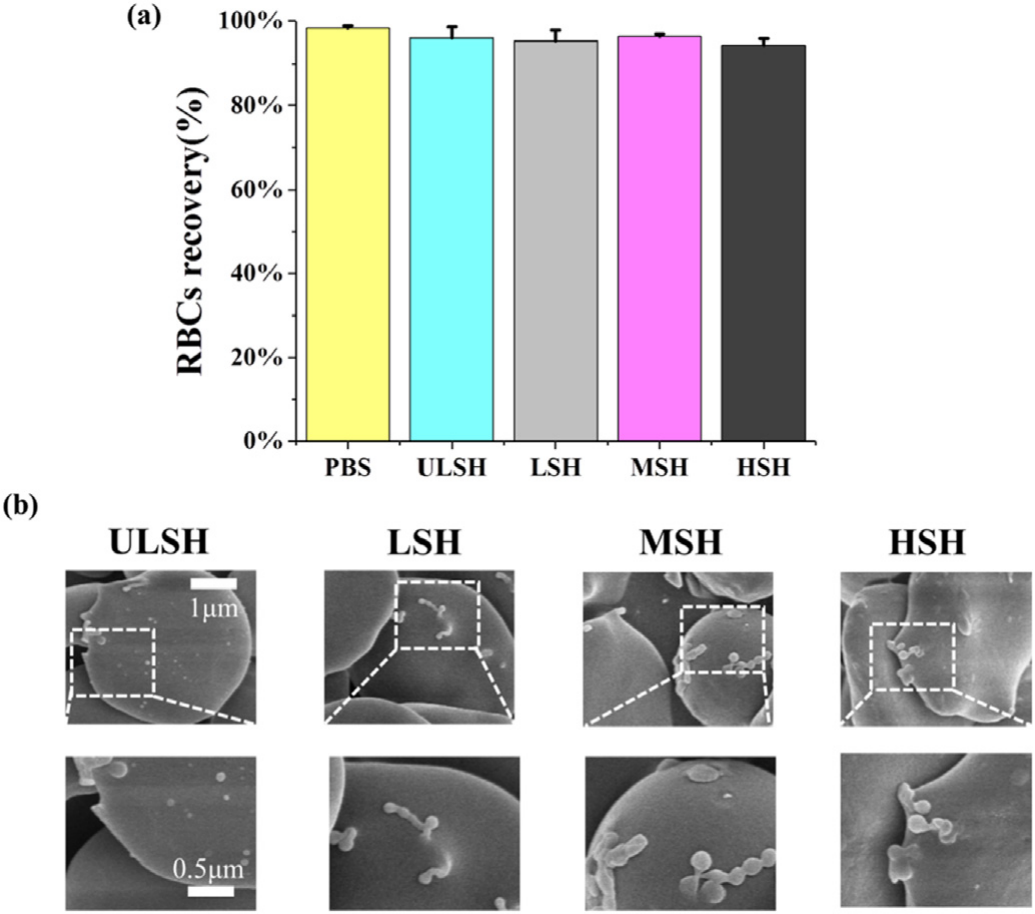
Biocompatibility test of SH. The recovery(a) and SEM images (b) of RBCs incubated in SH solutions at 4 ​°C for 24 ​h, PBS was used as a negative control. HSH, MSH, LSH and ULSH stand for high, medium, low and ultra-low molecular weight SH, respectively. The concentration of all SH groups was 1 ​mg/mL. Three replicates of each sample were tested and the data were shown as mean ​± ​SD.

To investigate whether SH caused severe hemolysis, different molecular weights of SH (HSH, MSH, LSH and ULSH) were prepared into 1 ​mg/mL. Then RBCs were added to PBS and SH groups and incubated at 4 ​°C for 24 ​h. The results were shown in Fig. 2a. The RBCs recovery of HSH group was the lowest (94.3 ​± ​1.5%), while that of other groups was all above 95%, indicating SH had good biocompatibility. Since the viscosity and water binding capacity of SH are positively correlated with molecular weight, HSH may dehydrate RBCs remarkably and lead to relatively high hemolysis.

To further discuss the reason for the good biocompatibility of SH, the interaction between RBCs and SH was determined through SEM (Fig. 2b). The RBCs membranes were attached with SH, and RBCs were maintained normally without obvious deformation or rupture. In particular, the RBCs in HSH group showed relatively serious dehydration. The results indicated that SH and RBCs could coexist successfully, and explained the reason for low hemolysis during incubation and confirmed the biocompatibility of SH.

### 3.2 Cryopreservation of RBCs

**Fig. 3:**
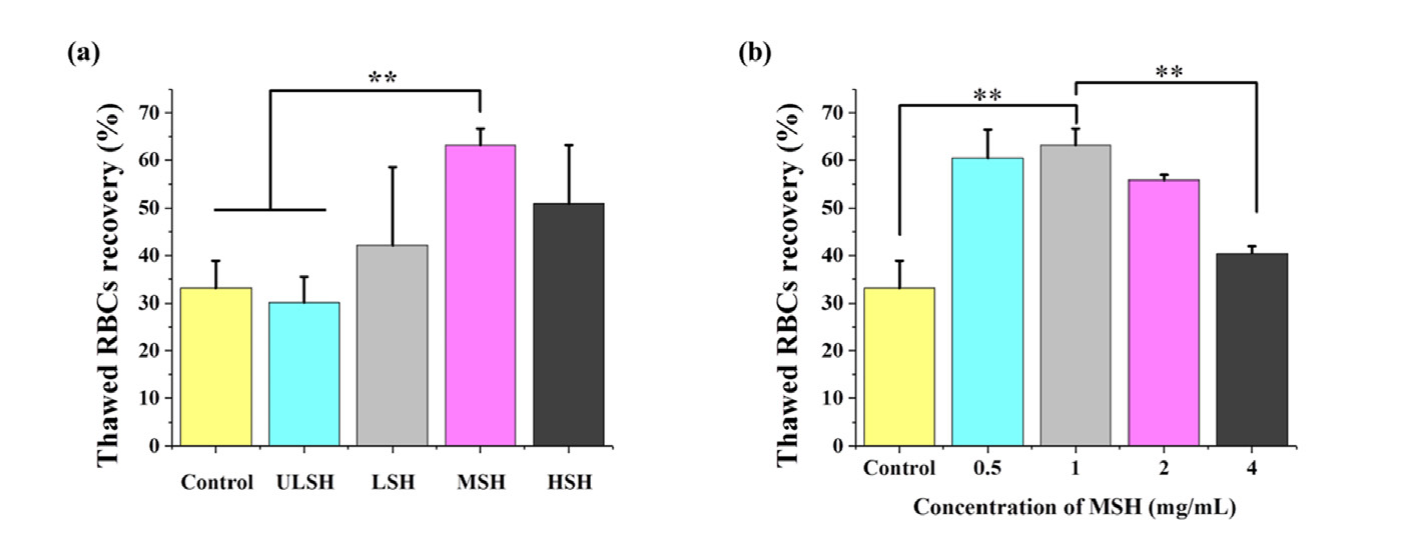
Thawed RBCs recovery with (a) different molecular weight SH and (b) different concentrations of MSH. The control group was PBS. Three replicates of each sample were tested and the data were shown as mean ​± ​SD. ∗∗ indicates *p* ​< ​0.01.

Due to the good biocompatibility, we explored the feasibility of SH in cryopreservation using fast freezing. The thawed recovery of RBCs treated with different SH (1 ​mg/mL) is shown in Fig. 3a. In MSH group the thawed RBCs recovery was highest (63.2 ​± ​3.5%), which was significantly different from the control group (33.1 ​± ​5.8%, 0.01 ​M PBS) and ULSH group (30.2 ​± ​5.3%) (*p* ​< ​0.01). In contrast, The thawed RBCs recovery of LSH group (42.1 ​± ​16.4%) and HSH group (51.0 ​± ​12.2%) were improved slightly compared with the control group. Therefore, we used different concentrations of MSH (0.5, 1, 2, 4 ​mg/mL, respectively) for further experiments, and the result is shown in Fig. 3b. The thawed RBCs recovery of the 1 ​mg/mL group was still the highest and was not significantly different from that of the 0.5 ​mg/mL group (54.1 ​± ​6.1%) and 2 ​mg/mL group (57.3 ​± ​1.0%). However, the thawed RBCs recovery of the 4 ​mg/mL group was only 42.3 ​± ​1.5%, which was close to the control group. The water binding capacity of SH is positively correlated with molecular weight and concentration. Therefore, in high molecular weight or high concentration SH solutions, RBCs would be seriously osmotic dehydration and the thawed RBCs recovery could be decreased. However, when SH is at low concentration or low molecular weight, the water binding capacity is too weak and its performance in cryopreservation is not much different from PBS. MSH has moderate water binding capacity at the appropriate concentration (1 ​mg/mL) and can achieve the best cryopreservation efficiency.

### 3.3 The molecular weight of MSH

**Fig. 4:**
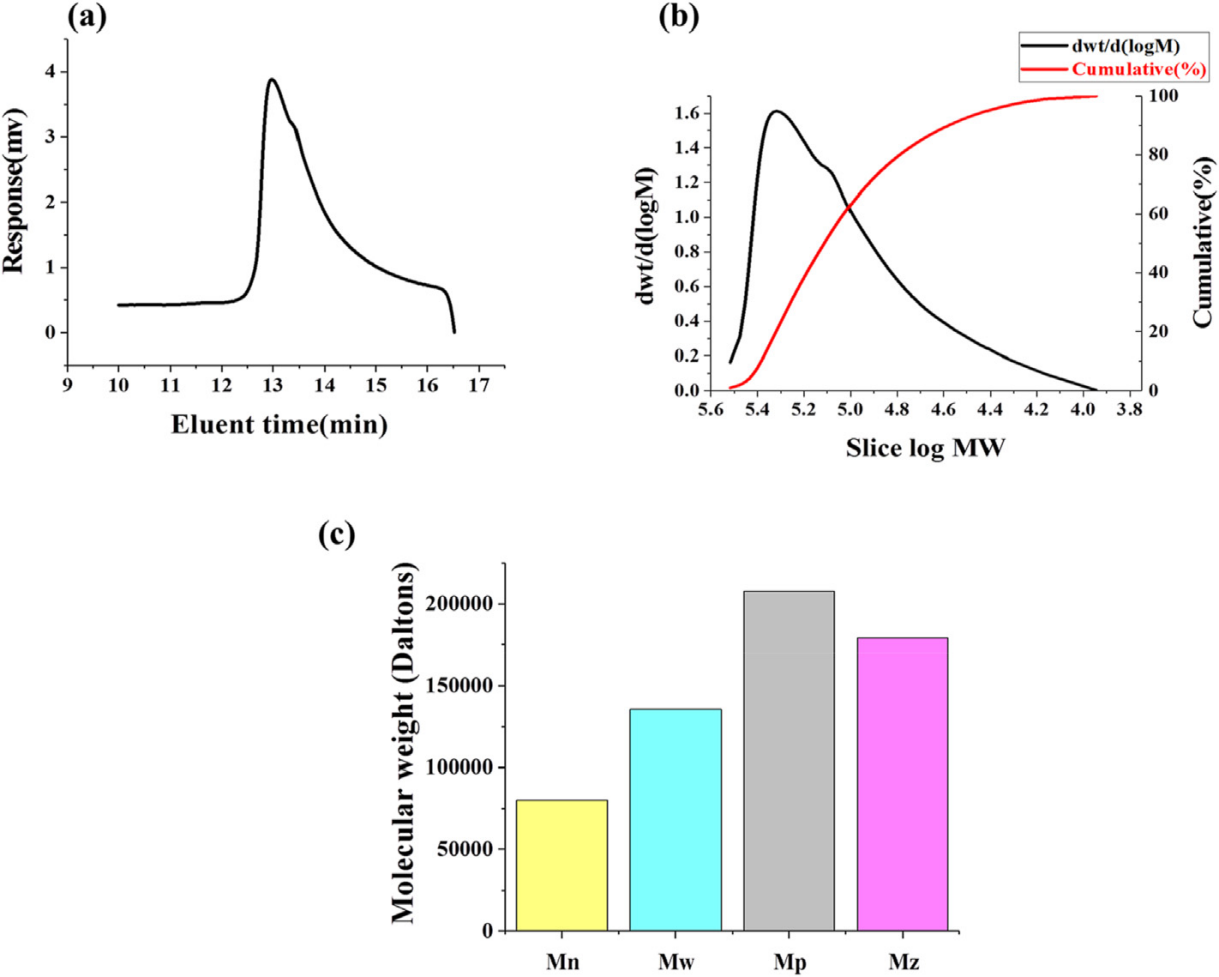
GPC chromatogram of MSH. (a) Elution time. (b) Molecular weight distribution. (c) Molecular weight with different definitions. Mn, Mw, Mp, and Mz are the number average molecular weight, weight average molecular weight, viscosity average molecular weight and z average molecular weight, respectively.

To study the mechanism of MSH in cryopreservation, GPC was used to determine the molecular weight distribution of MSH. The peak shape of the elution time was narrow, indicating that the molecular weight distribution of MSH was concentrated (Fig. 4a). The number average molecular weight (Mn) was 80008, the weight average molecular weight (Mw) was 135547, the viscosity average molecular weight (Mp) was 207909, the z average molecular weight (Mz) was 179144, and the PDI was 1.694 (Fig. 4b and c). Generally, the molecular weight of SH can reach more than 1 million. Using MSH with moderate molecular weight, the optimal viscosity and water binding capacity can be achieved, and the efficiency of cryopreservation can be enhanced.

### 3.4 Adjustment of osmotic pressure by “reservoir effect”

**Fig. 5:**
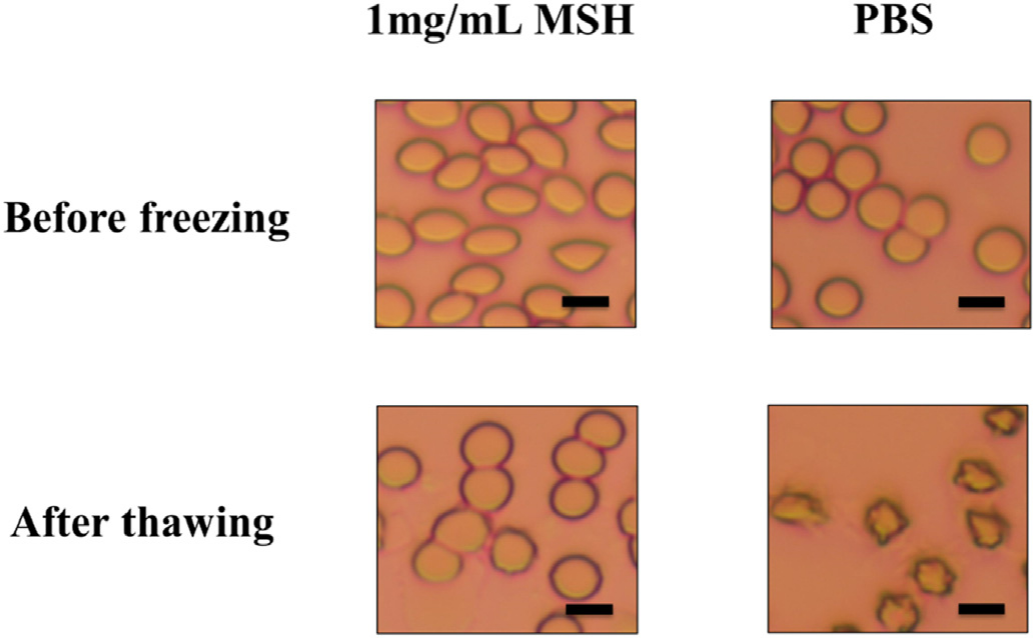
The morphology of RBCs. In different solutions (1 ​mg/mL or PBS) and different periods (before freezing and after thawing), the morphology has changed. Scale bar ​= ​5 ​μm.

To further determine the mechanism of MSH in cryopreservation of RBCs, the morphology of RBCs in different periods was observed. As shown in Fig. 5, RBCs incubated in 1 ​mg/mL MSH for 30min exhibited ellipsoidal shape before freezing, while RBCs in PBS showed the normal spherical shape. Because the strong water binding capacity of MSH led to the increase of osmotic pressure in the solution, and RBCs would change to ellipsoidal by partially osmotic dehydration. Therefore, the reduction of intracellular water in RBCs resulted in a decrease in intracellular ice content and damage during freezing. After thawing at a 37 ​°C water bath, the RBCs morphology in MSH group returned to the normal spherical, which was consistent with that of fresh RBCs. We hypothesized that most of the water generated from ice thawing was absorbed by MSH, reducing the osmotic impact on RBCs. In addition, MSH released part of the bound water during thawing, which slowly permeated into the RBCs to restore their normal morphology. However, the RBCs in PBS group were severely dehydrated which was caused by osmotic damage during freezing and thawing. In cryopreservation, MSH acted like a reservoir to periodically regulate the content of intracellular water of RBCs. It could reduce the cryoinjury to RBCs, and restore RBCs to the normal morphology after thawing. We named it the “reservoir effect”.

### 3.5 Ice recrystallization inhibition (IRI) activity

**Fig. 6:**
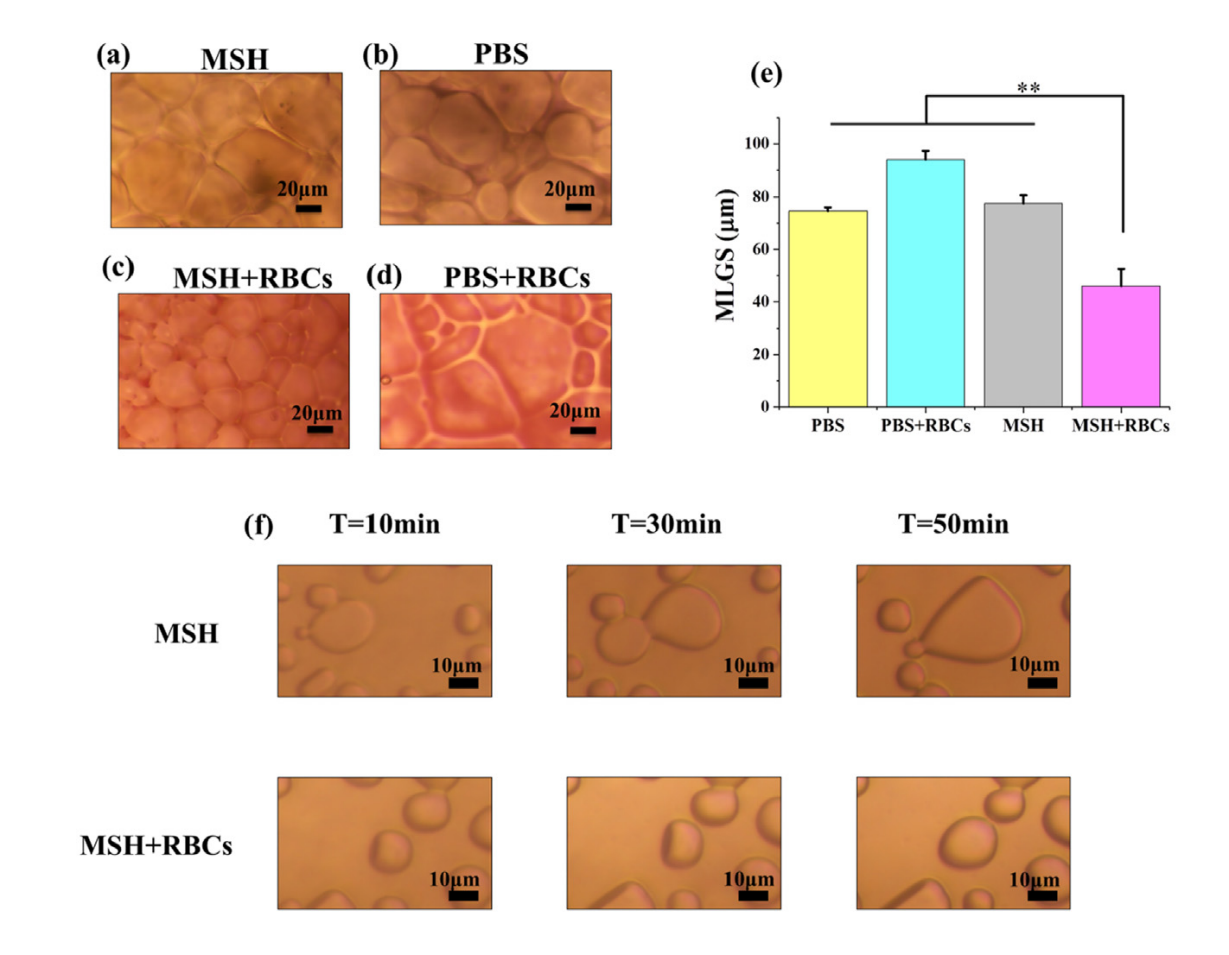
Ice recrystallization inhibition (IRI) activity. Microscopic images of ice crystals in the (a) MSH (1 ​mg/mL), (b) PBS, (c) MSH (1 ​mg/mL) +RBCs (8% hematocrit) and (d) PBS ​+ ​RBCs (8% hematocrit). (e) Quantitative analysis of IRI activity, MLGS ​= ​mean largest grain size. (f) Ice growth detected by sucrose sandwich assay. The microscopic images of ice growth in MSH group and MSH ​+ ​RBCs group (8% hematocrit) at 10min, 30min and 50min. Three replicates of each sample were tested and the data were shown as mean ​± ​SD. ∗∗ indicates *p* ​< ​0.01.

Splat assay was used to explore the ice recrystallization inhibition (IRI) activity of MSH. During the thawing process, small ice crystals will disappear and large ice crystals will grow up. It is known as ice recrystallization and is in line with the Ostwald ripening [37]. The large ice crystals formed by recrystallization are fatal to cells. Therefore, antifreeze proteins [38], polyvinyl alcohol [13], metal-organic framework [12] and other materials with IRI activity have the potential to become CPAs. As shown in Fig. 6a and b, there was no significant difference in the size of ice crystals between the PBS group and the MSH group. After the addition of RBCs to give a hematocrit content of 8%, the ice crystal size was significantly decreased in the MSH ​+ ​RBCs group while there was no obvious change in the PBS ​+ ​RBCs group (Fig. 6c and d). MLGS was used for quantitative analysis and the result was shown in Fig. 6e. The MLGS of MSH group and PBS group were 77.42 ​± ​3.17 ​μm and 74.62 ​± ​1.38 ​μm, respectively, and there was no significant difference between them. It showed that MSH could not reduce the size of ice crystals when used alone. However, the MLGS of the MSH ​+ ​RBCs group was only 45.98 ​± ​6.60 ​μm, which was obviously smaller than other groups. It indicated that when combined with RBCs, MSH could effectively inhibit ice recrystallization and reduce the mechanical damage from large ice crystals.

To further explore the IRI activity of the MSH ​+ ​RBCs group, the sucrose sandwich assay was used to observe the ice growth. Sucrose sandwich assay is to add enough sucrose to the sample to make its concentration reach 60%wt. The movement and fusion of ice crystals would be hindered by the high concentrations of sucrose, which facilitates the observation of ice growth under the microscope. As shown in Fig. 6f, from 10 ​min to 50 ​min, adjacent ice crystals got close to each other and gradually fused into one, leading to the increase of the size of ice. However, the ice growth in the MSH ​+ ​RBCs group was significantly slowed down, and the size of the ice crystal did not increase obviously from 10 ​min to 50 ​min. The whole process of ice growth could be seen in Supporting Information, Movie S1. It indicated that the combination of MSH and RBCs could effectively inhibit ice growth and further validated its IRI activity.

Supplementary data related to this article can be found at https://doi.org/10.1016/j.mtbio.2021.100156.

The following is the supplementary data related to this article:

Multimedia component 1 Multimedia component 1

**Fig. 7:**
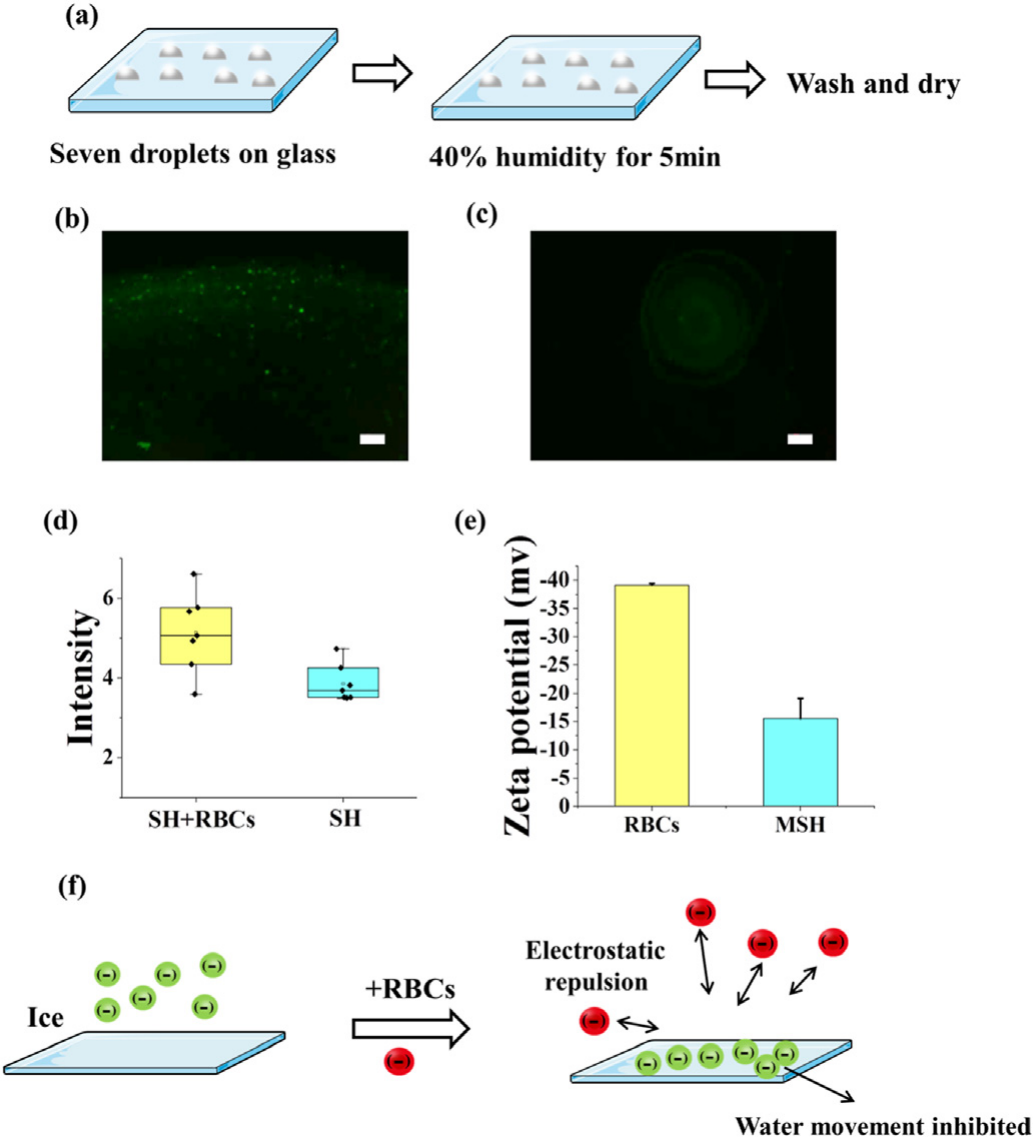
The electrostatic repulsion of RBCs and SH. (a) The schematic of the glass slide experiment. The fluorescence images of MSH ​+ ​RBCs(b) and SH group(c). (d) The fluorescence intensity of MSH ​+ ​RBCs and MSH group. (e) The zeta potential of RBCs and MSH. (f) The mechanism of the electrostatic repulsion between SH and RBCs. Scale bar ​= ​100 ​μm.

To study the mechanism of IRI activity of MSH, the glass slide experiments were operated (Fig. 7a) [31]. The fluorescence intensity of SH ​+ ​RBCs was obviously higher than that of SH alone (Fig. 7b–d), indicating more SH adhered to the surface. In addition, the zeta potential of SH and RBCs were all negative (Fig. 7e), so we hypothesized the SH would be forced to the surface by the electrostatic repulsion from RBCs (Fig. 7f). According to the previous study, the water movement among ice grains underlies ice recrystallization [39]. The SH adhered in the ice surface might inhibit the water movement due to its strong water binding capacity and high viscosity, and it might be the reason for the IRI activity. It must be noted that the electrostatic repulsion effect was just a hypothetical mechanism. The true mechanism of IRI activity of the combination of MSH and RBCs was still unclear, and further study was needed to determine it.

### 3.6 Reduction of the content of ice

**Fig. 8:**
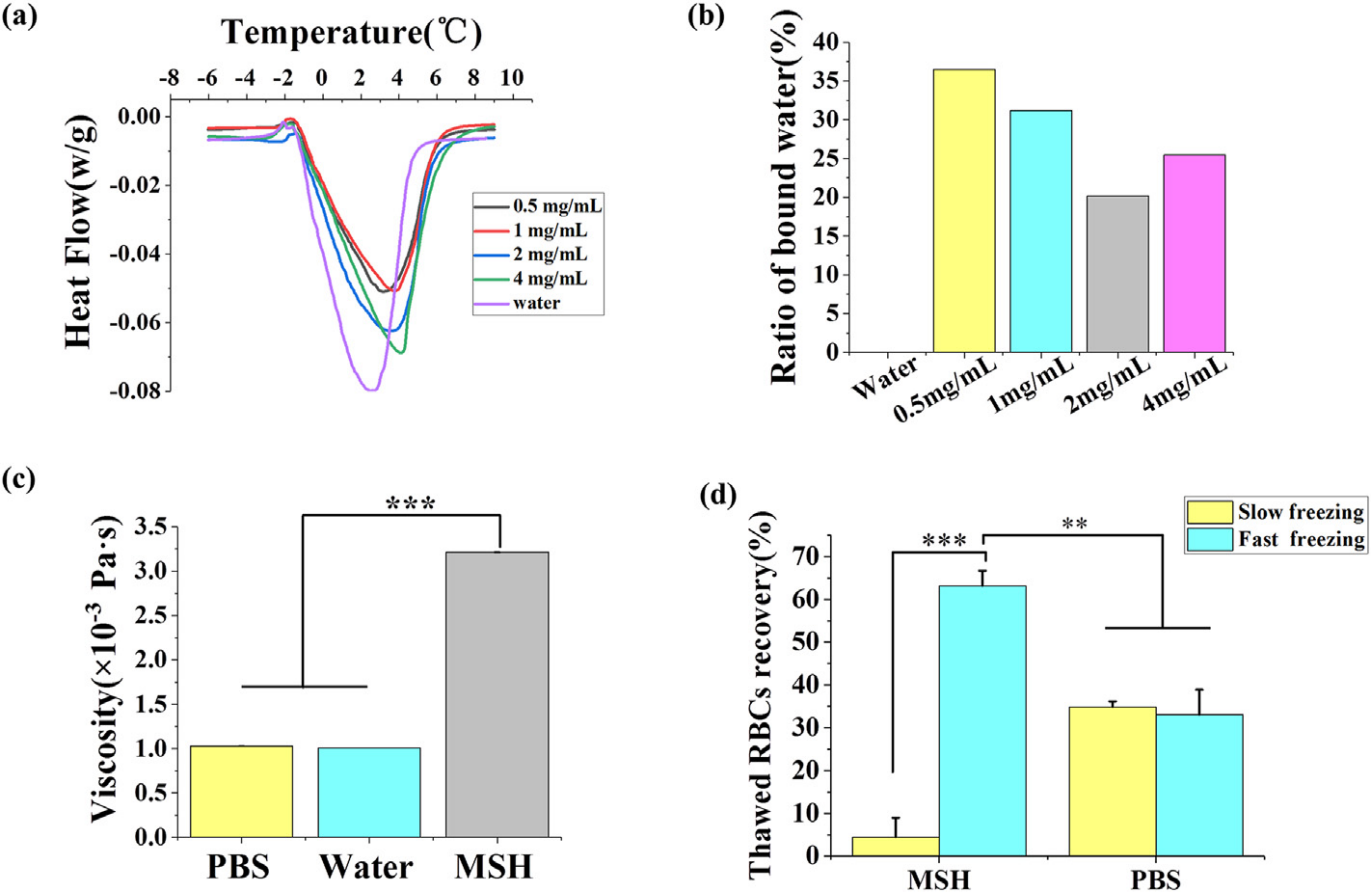
Effect of MSH on the content of ice. (a) The heat flow during the melting process and (b) ratio of bound water of pure water and different concentrations of MSH. (c) The viscosity of PBS, pure water and MSH. (d) The thawed RBCs recovery in PBS group and MSH group through fast freezing and slow freezing. Three replicates of each sample were tested and the data were shown as mean ​± ​SD. ∗∗ indicates *p* ​< ​0.01, ∗∗∗ indicates *p* ​< ​0.001.

DSC was used to determine the ratio of bound water of the samples during the freezing-thawing process. As shown in Fig. 8a, the endothermic peak area of pure water was obviously larger than that of MSH group. It indicated that there was bound water in MSH group which was difficult to freeze at low temperature, and the bound water was advantageous for reducing the content and damage of ice. The ratio of bound water in 0.05 ​mg/mL and 1 ​mg/mL MSH group was 36.5% and 31.2%, respectively, which indicated that MSH could produce more bound water at a lower concentration (Fig. 8b). In contrast, the ratio of bound water was significantly reduced in the 2 ​mg/mL and 4 ​mg/mL MSH groups. It was possible that at higher concentrations, the viscosity of MSH would increase significantly, and the molecules would entangle with each other to form a network structure. Therefore the water binding capacity was weakened.

Vitrification allows water molecules to remain in the disordered state of a liquid, but physical properties become more similar to those of a solid. Vitrification is important for cryopreservation because the natural state of water in organisms is disordered. Maintaining the disordered state of water molecules is conducive to minimizing the interference with the samples in cryopreservation. Moreover, vitrification makes liquid become the “solid liquid” rather than ice, which reduces the content and damages of ice [40]. Previous studies have pointed out that high cooling rates and high viscosity are beneficial to vitrification. First, when the temperature falls below the glass transition temperature (T_g_) at a sufficient rate, ice is difficult to form or grow obviously. Second, the high viscosity delays intermolecular rearrangements to inhibit ice forming [41]. Therefore, we studied the effect of viscosity and cooling rate on the thawed RBCs recovery to determine whether MSH acts through vitrification in cryopreservation. As shown in Fig. 8c, the viscosity of 1 ​mg/mL MSH was 3.216 ​± ​0.007 ​× ​10^−3^ ​Pa ​s, which was obviously higher than that of PBS (1.030 ​± ​0.004 ​× ​10^−3^ ​Pa ​s) and pure water (1.002 ​× ​10^−3^ ​Pa ​s) (*p* ​< ​0.001). For different cooling rates, as shown in Fig. 8d, there was no significant difference in thawed RBCs recovery between slow freezing (34.9 ​± ​1.3%) and fast freezing (33.1 ​± ​5.8%) in the PBS group. Because increasing the cooling rate alone might not promote vitrification when the viscosity was insufficient. However, the thawed RBCs recovery of MSH group through slow freezing was only 4.5 ​± ​4.3%, which was significantly different from that through fast freezing (63.2% ​± ​3.5%) (*p* ​< ​0.001). It indicated that MSH might improve the thawed RBCs recovery by promoting vitrification in fast freezing. And the large number of ice crystals formed by slow freezing would exceed the regulatory capacity of MSH, and cause adverse effects due to serious osmotic dehydration and mechanical damage. It must be pointed out that we only verified from the side that MSH can promote vitrification, and more experiments were needed to prove it thoroughly.

In summary, MSH could increase the ratio of bound water and reduce the formation of ice crystals during freezing. Additionally, the high viscosity of MSH could promote vitrification under fast freezing, and the water would become “solid liquid” instead of ice crystals. Therefore the damages from ice crystals were reduced and the thawed RBCs recovery was improved.

### 3.7 Direct washing of RBCs

**Fig. 9:**
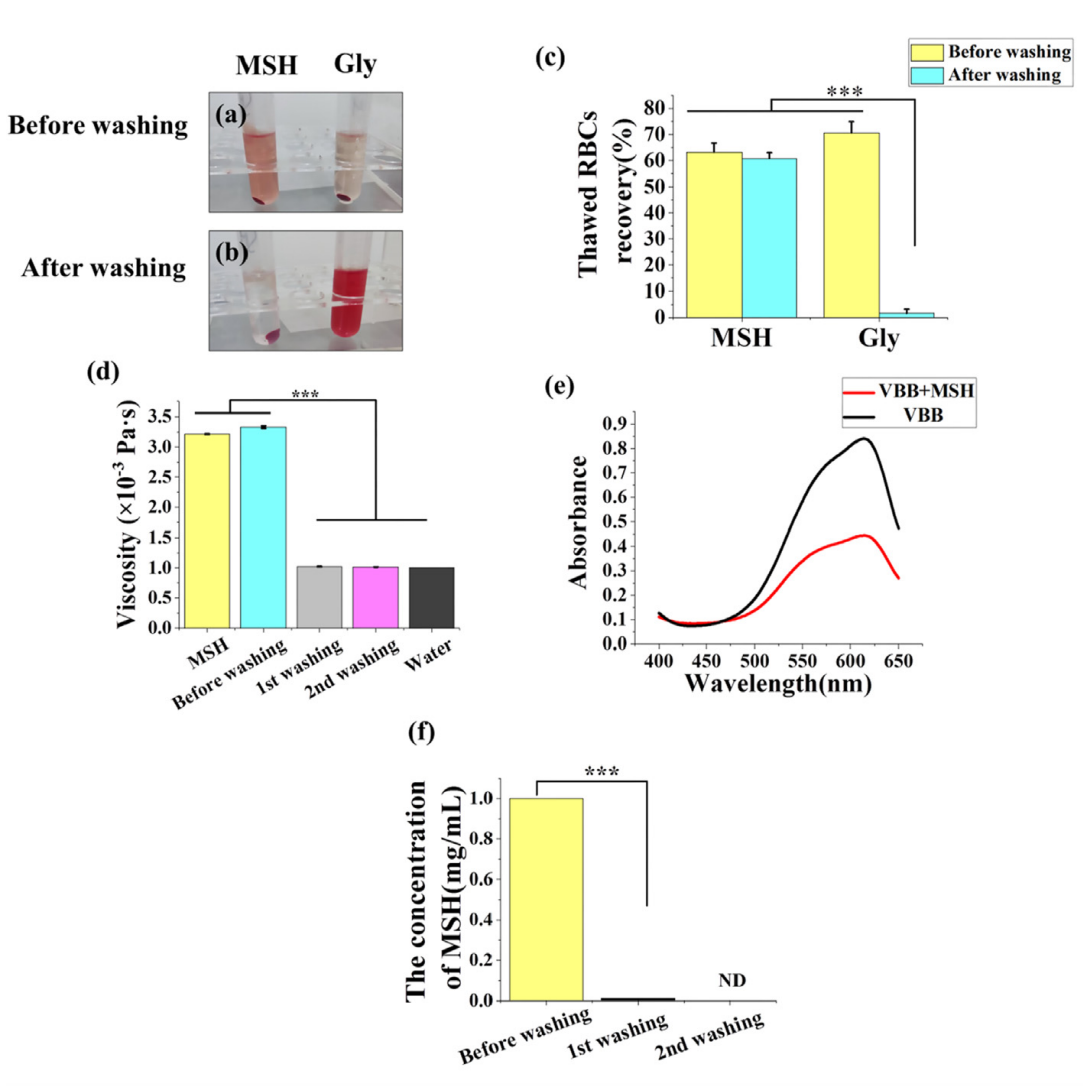
Direct washing of RBCs. The images of MSH group and Gly group before (a) and after (b) washing. (c) The thawed RBCs recovery before and after washing in different groups. (d) The viscosity of the MSH group before and after washing, and that of 1 ​mg/mL MSH and pure water as a control. (e) The UV–Vis spectra of VBB and VBB ​+ ​MSH, and the maximum absorption wavelength is 617 ​nm. (f) The efficiency of MSH removal in direct washing. The MSH concentration before washing is 1 ​mg/mL. Three replicates of each sample were tested and the data were shown as mean ​± ​SD. ∗∗∗ indicates *p* ​< ​0.001. ND=Not detected.

When cryopreserved RBCs are used for transfusion, the residual CPAs must be completely removed [42]. However, as a common CPA, Gly can change the osmotic pressure of RBCs. Therefore Gly in RBCs must be removed through multiple and complicated washing steps, which increases the cost and further causes hemolysis [43]. Herein, we attempted to directly wash the thawed RBCs that were treated with MSH and Gly and to explore changes in thawed RBCs recovery and CPA residues. As shown in Fig. 9a–c, before washing there was no significant difference in thawed RBCs recovery between MSH group and Gly group (63.2% ​± ​3.5% and 70.6 ​± ​4.3%, respectively). After washing, the thawed RBCs recovery of MSH group decreased slightly to 60.7 ​± ​2.3%. However, severe hemolysis occurred in the Gly group, and thawed RBCs recovery was only 1.7 ​± ​1.5%, which was significantly different from other groups (*p* ​< ​0.001). MSH could adjust the intracellular water content through the reservoir effect, so the osmotic shock during the washing process could be avoided. On the contrary, Gly permeated into RBCs and caused an increase in osmotic pressure. When washed directly, the RBCs membrane would rupture due to the huge osmotic shock. It was the reason for severe hemolysis and the dramatic decrease in thawed RBCs recovery.

To test the washing efficiency, the change of viscosity was analyzed (Fig. 9d). Before washing the viscosity of MSH group was 3.331 ​± ​0.021 ​× ​10^−3^ ​Pa ​s, which even exceeded that of 1 ​mg/mL MSH (3.216 ​± ​0.007 ​× ​10^−3^ ​Pa ​s). Because the sample contained not only MSH, but also RBCs fragments and other substances. After the first washing, the viscosity decreased dramatically to 1.022 ​± ​0.006 ​× ​10^−3^ ​Pa ​s, which was not much different from that of pure water (1.002 ​× ​10^−3^ ​Pa ​s). It indicated that the residual RBCs fragments and MSH have been basically removed from the sample. After the second washing, the viscosity was 1.015 ​± ​0.002 ​× ​10^−3^ ​Pa ​s, which was not far from the previous, indicating that the ideal washing efficiency could be achieved by the first washing. To further determine whether MSH was removed during washing, the fading spectrophotometry was used to study the concentration of MSH. VBB will fade when MSH was added, and the reduced absorbance ΔA is proportional to the MSH concentration [44]. The two samples all had the maximum absorbance at 617 ​nm and this wavelength was selected for detection (Fig. 9e). After the first washing, the concentration of MSH dropped sharply to 0.0110 ​± ​0.000 ​mg/mL, indicating that MSH has been almost completely removed. After the second washing MSH was not detected (Fig. 9f). In summary, RBCs cryopreserved by MSH could completely remove CPA and RBCs fragments by only once direct washing, while the thawed RBCs recovery would not change greatly. The convenient washing step is very meaningful for clinical transfusion treatment.

### 3.8 The properties and functions of the washed RBCs after cryopreservation

**Fig. 10:**
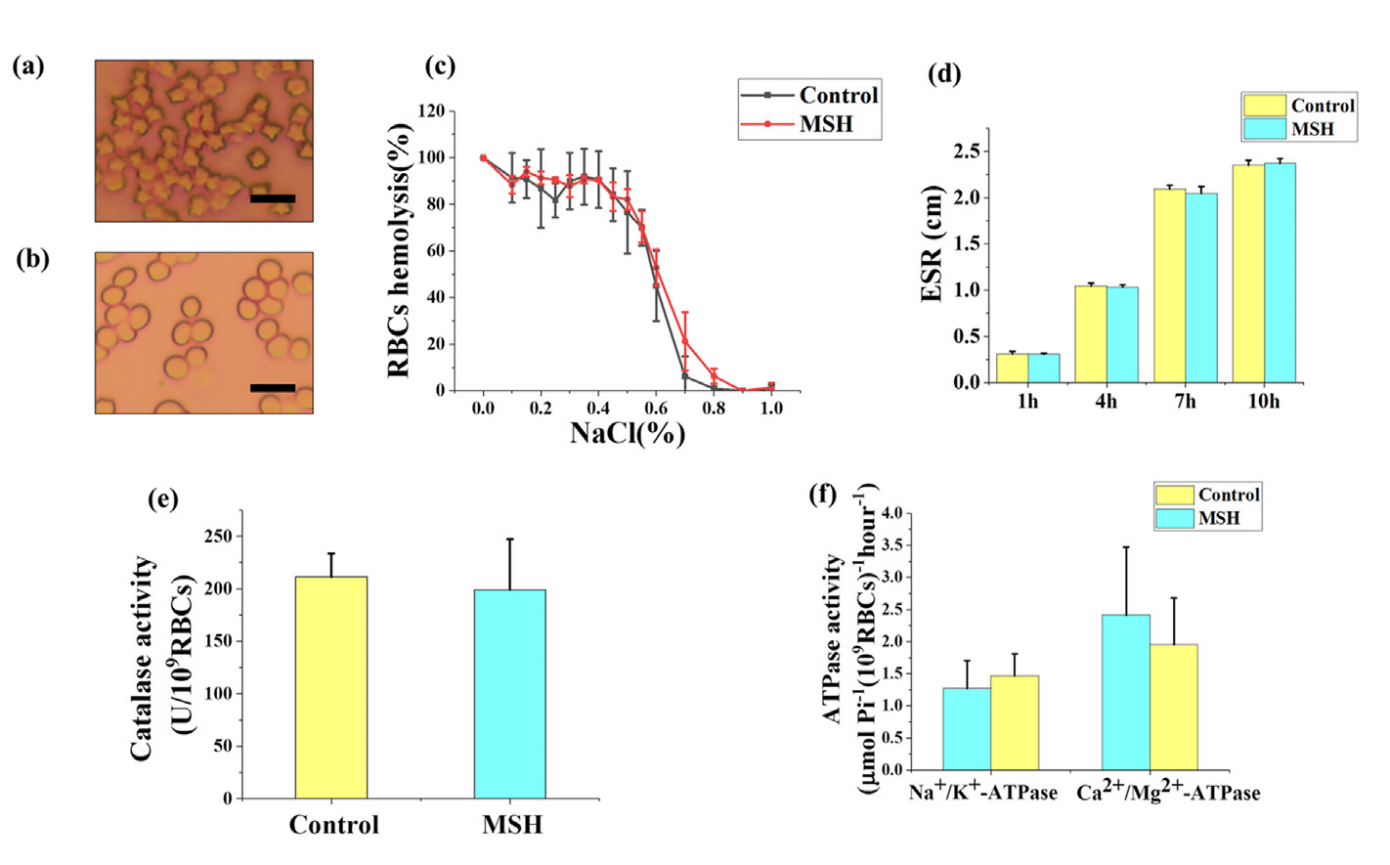
The properties and functions of washed RBCs after cryopreservation. The morphology of washed RBCs after cryopreservation in PBS group (a) and 1 ​mg/mL MSH group (b). The osmotic fragility (c), ESR (d), catalase activity (e) and ATPases activities (f) of washed RBCs after cryopreservation in 1 ​mg/mL MSH group and control group (fresh RBCs). ESR ​= ​erythrocyte sedimentation rate. Three replicates of each sample were tested and the data were shown as mean ​± ​SD. Scale bar ​= ​10 ​μm.

Whether RBCs can maintain normal properties and functions is the key to the success of transfusion therapy. Herein we have tested the functions and properties of washed RBCs after cryopreservation from different perspectives. The morphology of RBCs in PBS group, as shown in Fig. 10a, was severely dehydrated and became thorny due to the osmotic and mechanical damages during cryopreservation. However, the washed RBCs after cryopreservation maintained the normal spherical shape in the MSH group (Fig. 10b). The osmotic fragility reflects the membrane stability of RBCs. As shown in Fig. 10c, the 50% hemolysis happened at about 0.6% NaCl solutions both in the MSH and control group. The result showed the membrane stability of washed RBCs after cryopreservation in MSH group was not obviously changed. In addition, ESR fluctuates within a narrow range in normal RBCs, and the huge increment of ESR indicated the properties of RBCs have been damaged. As shown in Fig. 10d, within 1, 4, 7, and 10 ​h, there was no significant difference in ESR between the MSH group and the control group. Additionally, catalase can degrade hydroxyl radical (OH·), which is considered to be the most active ROS and can destroy lots of intracellular organic material. The activity of catalase between MSH and the control group was not obviously different, indicating the biochemical property of washed RBCs after cryopreservation was maintained (Fig. 10e). Moreover, the ATPases, including Na^+^/K^+^ ATPase and Ca^2+^/Mg^2+^ ATPase, are important enzymes in the RBCs membrane and can maintain the intracellular gradients of ions. Both the Na^+^/K^+^ ATPase and Ca^2+^/Mg^2+^ ATPase activities of the MSH group were not significantly different from the control group (Fig. 10f).

In summary, six indexes of RBCs properties and functions, including morphology, ESR, Na^+^/K^+^ ATPase activity, Ca^2+^/Mg^2+^ ATPase activity, catalase activity and osmotic fragility were tested, and all of the indexes of washed RBCs after cryopreservation treated with MSH were maintained normally. As discussed above, MSH could regulate the intracellular water content by reservoir effect, inhibit ice recrystallization when combined with RBCs, increase the ratio of bound water and promote vitrification. Therefore the damages during freezing, thawing and washing were reduced and RBCs could maintain their normal functions and properties. We believe the washed RBCs after cryopreservation can work properly and can be used in transfusion in the clinic.

### 3.9 Improve the efficiency of cryopreservation

**Fig. 11:**
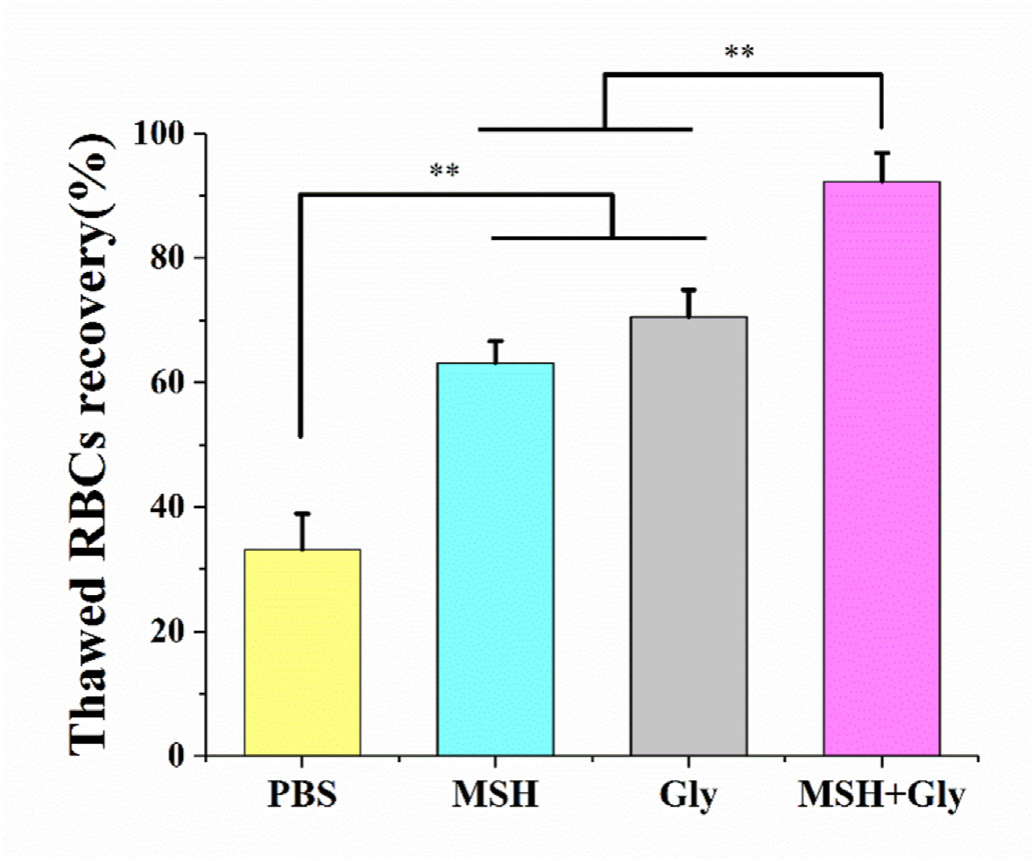
Thawed RBCs recovery when MSH and Gly were used alone or in combination. The MSH was 1 ​mg/mL, and Gly was 5%v/v. Three replicates of each sample were tested and the data were shown as mean ​± ​SD. ∗∗ indicates *p* ​< ​0.01.

To further improve the efficiency of cryopreservation for RBCs, MSH was used with a very low concentration of Gly (5%v/v) and the result was shown in Fig. 11. The combination of 1 ​mg/mL MSH+5%v/v Gly resulted in the thawed RBCs recovery as high as 92.3 ​± ​4.6%, which was significantly different from that of the 1 ​mg/mL MSH group and 5%v/v group (*p* ​< ​0.01). In general, 20–40% v/v Gly is required to achieve more than 90% thawed RBCs recovery. Therefore, the combination of 1 ​mg/mL MSH with 5% v/v Gly could reduce the damages to the RBCs from high concentrations of organic solvents and showed strong cryopreservation efficiency. Because in the incubation process before freezing, the MSH could partially dehydrate RBCs by reservoir effect. At the same time, Gly could permeate into RBCs to replace the residual intracellular water, which further reduced the generation of intracellular ice crystals during cryopreservation. In addition, Gly also had a high viscosity and could play a synergistic effect with MSH to promote vitrification. These effects together increased thawed RBCs recovery and demonstrated the powerful effect of MSH in cryopreservation.

### 3.10 Compare CPAs by the mathematical model

TOPSIS was used to quantitatively analyze the advantages of MSH compared to PVA and trehalose. As a classic mathematical model, TOPSIS is based on the concept that the chosen alternative should have the shortest distance from the positive ideal solutions (PIS) and the longest distance from the negative ideal solutions (NIS) [45]. In previous study trehalose and PVA were always used with other materials in cryopreservation (betaine and hydroxyethyl starch, respectively) [3,46], so the data of the combinations were chosen for analysis. The relevant criteria of the CPAs, such as price, optimal concentrations, thawed RBCs recovery, and the price/concentrations of the combination materials were collected to form an initial decision matrix, as shown in Table 1. The thawed RBCs recovery was determined to be the benefit attribute while others were the cost attributes. And the closeness coefficient and rank of the three CPAs were shown in Table 2. MSH had the highest closeness coefficient with 0.645, while PVA and trehalose were 0.539 and 0.388, respectively. It showed that MSH had the best efficiency under the five criteria, and indicated the bright prospect of MSH in cryopreservation.

**Table 1:** Three alterbative CPAs and five criteria.

Types of CPAs	Price (USD/g)^a^	Optimal concentrations (%wt)	Thawes RBCs recovery (%)	Price of combination agent (USD/g)^b^	Concentrations of combination agent (mg/mL)^*c*^	Reference
Trehalose	0.993	4.0	89.45	1.384	150	[3]
PVA	1.868	0.5	75.00	0.176	175	[46]
MSH	8.066	0.1	92.30	0.242	63	/

a The data of price were from the company websites that the reference had noted. All of them have been converted into USD/g for easy calculation.

b The combination agents were betaine, HES and Gly, respectively. The specification of Gly was given as volume (mL) on the company website, and it has been calculated to USD/g with the density of 1.261 ​g/mL ^*c*^The concentrations were uniformly converted by mg/mL.

**Table 2:** The result of TOPSIS.

Types of CPAs	Closeness coefficient	Rank
Trehalose	0.388	3
PVA	0.539	2
MSH	0.645	1

### 3.11 Proposed mechanism

**Fig. 12:**
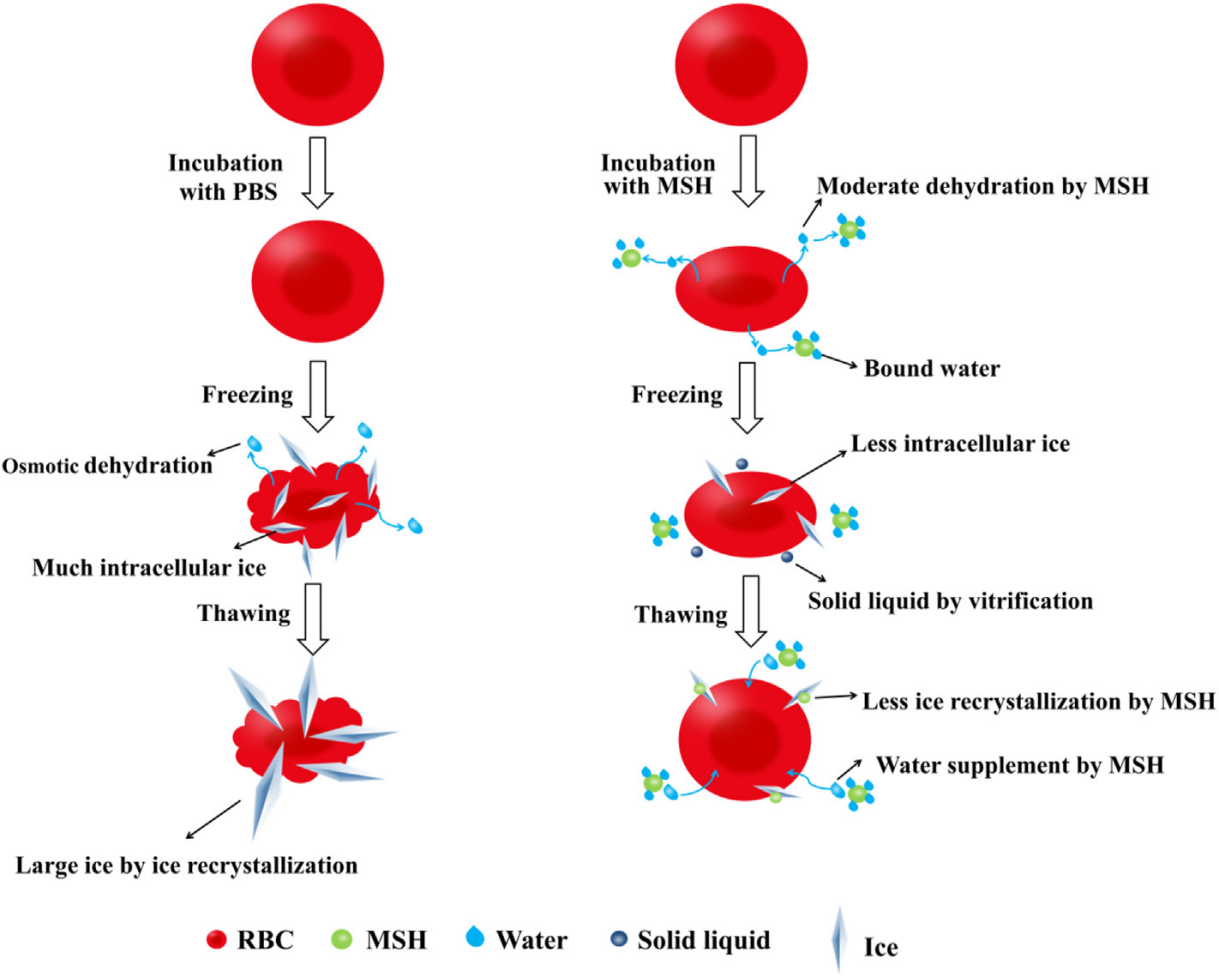
The mechanism of MSH in cryopreservation of RBCs.

Based on the above experiments, we proposed the mechanism of MSH in cryopreservation, as shown in Fig. 12. RBCs incubated with PBS would undergo severe osmotic dehydration and plenty of ice crystals would be formed. During thawing, the intracellular and extracellular ice would recrystallize and grow, further puncturing the cell membrane. These are the reasons for the low thawed RBCs recovery in PBS. When incubated with MSH, the RBCs would be moderately dehydrated and changed from spherical to ellipsoid due to the reservoir effect, which could reduce the content of intracellular ice during freezing. In addition, the high viscosity of MSH was conducive to vitrification, so the water would form a “solid liquid” instead of ice crystals. Due to the strong water binding capacity of MSH, plenty of free water would also become bound water which was difficult to freeze. These effects all reduced the content of intracellular and extracellular ice during freezing, which was beneficial for reducing mechanical and osmotic damage caused by ice. During thawing, the IRI activity of MSH could be activated by RBCs. Under the repulsion of the same charge of RBCs, MSH would approach the surface of the ice and inhibit the flow of water around the ice crystals through its strong water binding capacity. Therefore, ice recrystallization was inhibited and the mechanical damage of large ice to RBCs was reduced. In summary, MSH periodically regulated the water content of RBCs, promoted vitrification and increased the ratio of bound water. These could reduce the content of ice during freezing. When combined with RBCs, MSH could inhibit ice recrystallization during thawing. Therefore, osmotic and mechanical damages were reduced by these effects and the thawed RBCs recovery was improved.

## 4 Conclusions

MSH showed great potential in the cryopreservation of RBCs. As a biocompatible agent, MSH did not cause additional hemolysis. In cryopreservation, MSH could regulate the water content of RBCs through reservoir effect, increase the ratio of bound water, promote vitrification, and inhibit ice recrystallization when combined with RBCs. After thawing, MSH and other substances in RBCs could be removed with almost no additional hemolysis by direct washing. RBCs morphology and ESR remained normal after washing. In addition, MSH used with very low concentration glycerol (5% v/v) could improve the thawed RBCs recovery to over 90%. And according to the mathematical model analysis, the efficiency of MSH was better than that of trehalose and PVA. The whole process of cryopreservation was operable and clinically practical, which could solve the bottleneck of the short storage time of RBCs and promote transfusion therapy. This work also opened up new ideas for exploring the mechanism of CPAs, which might revolutionize the research and application of cryopreservation.

## Author contribution

Xiangjian Liu: Conceptualization, Investigation, Writing – original draft. Yuying Hu: Visualization, Formal analysis. Yuxin Pan: Validation. Meirong Fang: Methodology. Zhen Tong: Supervision. Yilan Sun: Resources. Songwen Tan: Writing – review & editing, Project administration

## Funding

This research did not receive any specific grant from funding agencies in the public, commercial, or not-for-profit sectors.

## Data availability

All data used to support the findings of this study are included within the article.

## Declaration of competing interest

The authors declare no conflict of interest.
